# Defining Fatty Acid Changes Linked to Rumen Development, Weaning and Growth in Holstein-Friesian Heifers

**DOI:** 10.3390/metabo12050374

**Published:** 2022-04-20

**Authors:** Emma N. Taylor, Jiwan Han, Congying Fan, Manfred Beckmann, Glyn Hewinson, David Rooke, Ad P. Koets, Luis A. J. Mur

**Affiliations:** 1 Institute of Biological, Environmental and Rural Sciences (IBERS), Aberystwyth University, Aberystwyth SY23 3DA, UK; emt26@aber.ac.uk (E.N.T.); meb@aber.ac.uk (M.B.); glh14@aber.ac.uk (G.H.); 2 College of Software, Shanxi Agricultural University, Taigu District, Jinzhong 030810, China; hanjiwan@sxau.edu.cn (J.H.); ttf300@126.com (C.F.); 3 Centre of Excellence for Bovine Tuberculosis, Aberystwyth University, Aberystwyth SY23 3DA, UK; 4 ProTEM Services Ltd., Horsham RH12 4BD, UK; david.rooke@dynamicextractions.com; 5 Wageningen Bioveterinary Research, 8221 RA Lelystad, The Netherlands; 6 Population Health Systems, Faculty of Veterinary Medicine, Utrecht University, 3584 CS Utrecht, The Netherlands

**Keywords:** metabolomics, fatty acids, eicosanoids, rumen development, weaning, growth

## Abstract

After birth, as effectively monogastric animals, calves undergo substantial physiological changes to become ruminants by 3 months of age and reach sexual maturity at approximately 15 months of age. Herein, we assess longitudinal metabolomic changes in Holstein-Friesian (HF) heifers from birth until sexual maturity during this developmental process. Sera from 20 healthy, HF heifers were sampled biweekly from 2 weeks of age until 13 months of age and then monthly until 19 months of age. Sera were assessed using flow infusion electrospray high-resolution mass spectrometry (FIE-HRMS) on a Q Exactive hybrid quadrupole-Orbitrap mass spectrometer for high-throughput, sensitive, non-targeted metabolite fingerprinting. Partial least squares discriminant analysis (PLS-DA) and unsupervised hierarchical clustering analysis (HCA) of the derived metabolomes indicated changes detectable in heifers’ sera over time. Time series analyses identified 30 metabolites that could be related to rumen development and weaning at ~3 months of age. Further time series analysis identified 40 metabolites that could be correlated with growth. These findings highlight the role of acetic acid and 3-phenylpropionate (3-PP) in rumen development and growth, suggest that weaning induces elevated levels of fatty acyls in response to a post-weaning stress-induced innate immune response and demonstrate the utilization of fatty acyls in growth. The identified metabolites offer serum metabolites which could inform the nutrition and healthy development of heifers.

## 1. Introduction

From birth to sexual maturity, dairy heifers exhibit substantial physiological and biochemical changes associated with rumen development, weaning and growth. Prior to the development of a functional rumen at approximately 3 months, calves are effectively monogastric, as the esophageal groove ensures that liquid feeds are directed to the abomasum and small intestine [1]. The development of the rumen allows heifers to convert complex plant carbohydrates into simple sugars via fermentation by rumen microbes. These simple sugars are later converted into volatile fatty acids (VFAs), such as acetate, propionate and butytrate, which are used as energy sources in heifer maintenance, growth and pregnancy [2].

Following a typical birth weight of 43.4 kg (±4.93) and weaning weight of 69.8 kg (±8.38) [3], dairy heifers demonstrate a typical growth rate of 0.584 kg/day until 22 months of age [4]. Sexual maturity is reached at approximately 60% of their mature liveweight, which equates to ~330 kg at 15 months of age, based on a mature liveweight of 550 kg [5]. Heifer growth rates influence the financial sustainability of dairy farms. Dairy heifers exhibit birth to first-calving rearing costs of £1819 ± 387/heifer (£2.31 ± 0.41/day/heifer) and each heifer which exceeds 784 days of age at first calving exhibits additional rearing costs of £2.87/day [6]. Thus, an in-depth metabolomic understanding of the biochemical pathways active during physiological changes, such as rumen development and weaning, could enable nutritionists and farmers to adapt and develop management strategies to improve herd health and productivity.

Many studies have examined factors that may influence rumen development and adaptation, such as weaning [7] and sexual maturity [8]. Rumen development is affected by liquid and starter feed, forage and physical feed types, volatile fatty acids (VFAs) and feeding management [7]. Growth is influenced by the type of dam (primiparous or multiparous), milk replacer availability and preweaning calf housing (individually or within groups) [8]. With regards to omics approaches, Li et al. [9] reviewed the use of omics approaches in examining milk yield and milk composition, the impacts of dietary treatments and identification of novel biomarkers for mastitis. Transcriptomic analysis of post-weaned beef heifers with varying feed efficiencies highlighted how more efficient heifers focused nutrients on growth and protein accretion as opposed to inflammation following exposure to a hepatic proinflammatory stimulus [10].

Given the nutritional–biochemical changes associated with heifer development, metabolomic approaches are particularly appropriate. Metabolomic techniques tend to focus on low-molecular weight compounds (<1 kD) often referred to as the end products of interactions between the genome and the environment. In the context of bovine research, the extensive characterization of the bovine metabolome is particularly useful. Thus, in bovine metabolomes for serum, ruminal fluid, the liver, longissimus thoracis muscle, semimembranosus muscle, and testis tissue, 51,801 bovine metabolites and 46,628 metabolites have been identified via genome-scale metabolite inference methods [11]. Some metabolomic assessments of lactating dairy cows have already been undertaken. Multi-omics approaches have successfully linked individual rumen microbial metabolites and specific microbial taxa to high milk protein yields [12] and milk-based saturated fatty acids [13]. In relation to rearing heifers, enhancing early-life nutrition affects energy, protein and liver metabolism [14], and also induces changes in mammary morphology and physiology-associated transcriptome expression [15]. However, metabolomic approaches have not been used to examine the changes linked to bovine weaning, rumen development and growth.

This study aimed to use time series analysis to determine the effects of age on the metabolomic profile of Holstein-Friesian (HF) heifers. Serum samples collected longitudinally from heifers between 2 weeks and 19 months of age were examined using flow infusion electrospray ionization high-resolution mass spectrometry (FIE-HRMS). We observed metabolomic changes correlated with rumen development and weaning followed by growth which are of likely functional relevance and indicative of heifer development.

## 2. Experimental Design

### Animal Samples

The animal experiments were approved by the Animal Welfare Body of Utrecht University (permit number 0202.0806) and in accordance with the Dutch regulations on animal experimentation. Sera from 20 healthy HF heifers were sampled biweekly from 2 weeks until 13 months of age and then monthly until 19 months of age. Liveweight (kg) data were estimated at approximately 30 days of age and biweekly from 3 months of age onwards based on chest circumference. Calves were raised following nutritional recommendations, i.e., calves had restricted access to a commercially available milk replacer and calf concentrate following the recommendations of the respective feed producers for rearing dairy calves.

1. Calves were born in May and kept indoors in small group accommodations (*n* = 4) on straw bedding. The calves had free access to hay and drinking water, in addition to restricted access to a commercially available milk replacer and calf concentrate.
2. Following weaning at ~6 weeks of age, the calves received a conventional diet based on grass silage and limited concentrates with free access to water.
3. From the beginning of July, the calves were housed in 2 groups of 10 but remained indoors on a ration of grass silage and limited concentrates with free access to water.
4. During the subsequent summer period (May until October), the heifers were kept on pasture with free access to grass and limited concentrates.
5. The heifers returned indoors from October until the end of the study.

Practices of animal husbandry during the experiment closely resembled dairy cattle farming practices commonly accepted in the Netherlands, including those of pasture grazing. Animals were bred at approximately 15 months of age. For logistical reasons, cows were synchronized for the start of the cycle using an ear implant for 9 days (3 mg Norgestomet, Crestar; Intervet International, Boxmeer, The Netherlands) accompanied by treatment with 3 mg Norgestomet with 5 mg estradiol valerate (i.m.; Intervet International). Two days before removal of the implant, prostaglandin (15 mg Prosolvin i.m.; Intervet International) was administered to ensure complete regression of the corpus luteum. It is known that the cows ovulate on average 3 days after removal of the Crestar ear implant, and this day was used for artificial insemination. Cows were subsequently monitored for oestrous behaviour and examined by rectal ultrasound pregnancy checks at 6 and 12 weeks following first (or repeat) insemination. Animals conceived between approximately 15 and 17 months of age.

## 3. Procedure

### 3.1. Untargeted Metabolite Fingerprinting by Flow Infusion Electrospray Ionization High-Resolution Mass Spectrometry (FIE-HRMS) 

Sera were prepared as described by Beckmann et al. [16], with minor amendments. Samples were defrosted on ice, vortexed for 5 s and 200 µL was pipetted into 1520 µL pre-chilled solvent mix (methanol/chloroform (4/1 *v*/*v*)) containing 1 micro-spoon of glass beads (<160 μM glass beads (Sigma-Aldrich Ltd., Gillingham, UK)). Samples were then vortexed for 5 s, shaken for 15 min at +4 °C and kept at −20 °C for 20 min. Following centrifugation at 21,000× *g* and 4 °C for 5 min, 100 µL of the plasma supernatant was transferred into mass spectrometry vials along with 100 µL methanol/water (70/30% *v*/*v*). Samples were stored at −80 °C until analysis using FIE-HRMS. For each sample, 20 µL were injected into a flow of 60 µL per minute in methanol/water (70/30% *v*/*v*), using a Surveyor flow system, into a Q Exactive plus mass analyser instrument with an ultra-high-performance liquid chromatography (UHPLC) system (Thermo Fisher Scientific©, Bremen, Germany) for high-throughput FIE-HRMS. Data acquisition for each serum sample was carried out using alternating positive and negative ionization modes, in four different scan ranges (15–110 *m*/*z*, 100–220 *m*/*z*, 210–510 *m*/*z*, 500–1200 *m*/*z*) with an acquisition time of 2 min. 

### 3.2. Statistical Analysis

Metabolomic data were analyzed using MetaboAnalyst 4 [17]. Data were subjected to interquartile range-based filtering, log10 transformations and Pareto scaling. Time series analyses, corrected for false discovery rate (FDR), were used to identify *m*/*z* ratios, which significantly (*p*-values < 0.05) differed between experimental classes. Major sources of variation were displayed using unsupervised hierarchical clustering analysis (HCA) and line graphs. Alternatively, variable importance in projection (VIP) scores were used to indicate *m*/*z* ratios which discriminated between the classes. VIP scores greater than 1 are typically used for selecting important variables. However, on occasion, VIP scores > 0.75 included in partial least squares discriminant analysis (PLS-DA) were shown to highlight variables with values close to 1.

Metabolites were identified using the DIMEdb database based on their *m*/*z* ratios, molecular formulae and the Bovine Metabolome Database [11]. All isotopes/adducts were considered. KEGG global metabolic network maps were based on KEGG molecular interaction networks [17]. Metabolite set enrichment analysis (MSEA) using over representation analysis (ORA) was used to highlight key pathways and biological patterns. Correlation analysis between metabolites and liveweight data (kg) was performed using Pearson’s correlation coefficient.

## 4. Results

**Figure 1:**
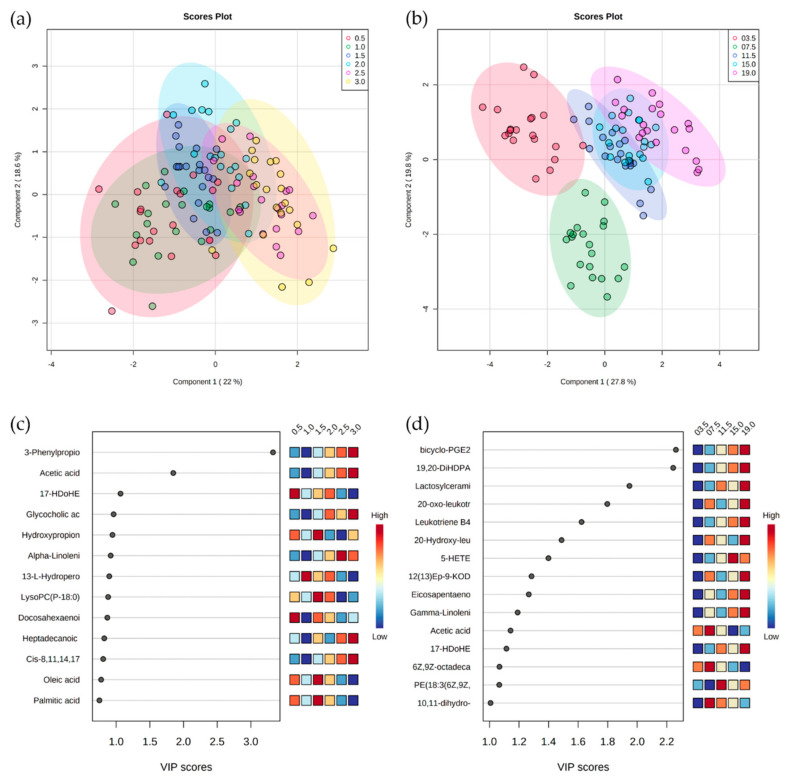
(**a**) PLS−DA of HF heifers between 0.5 and 3.0 months of age. (**b**) PLS−DA of HF heifers between 0.5 and 19.0 months of age. (**c**) VIP score plots produced by PLS−DA of metabolites with a VIP score >0.75 in HF heifers between 0.5 and 3.0 months of age to allow the visualization of metabolites whose values were close to 1. (**d**) VIP score plots produced by PLS−DA of metabolites >1.0 in HF heifers between 0.5 and 3.0 months of age. All in the combined ionization mode *m/z*.

PLS-DA assessments of sera derived from 20 healthy HF heifers sampled from 2 weeks until 19 months of age suggest gradual changes in the metabolome, although there appeared to be distinct phases. These were divided into two phases that broadly corresponded to pre- (3 months) and post-rumen development (>3 months) phases [2] (Figure S1). The metabolomes did not change during the first month after birth but there was a shift towards a distinct metabolome that was established at 2.5 and 3 months, with subsequent changes probably reflecting increases in rumen muscle mass and rumen papillae development (Figure 1a). Considering later time points, metabolomic changes continued until 11.5 months, after which all the serum metabolomes appeared to be similar (Figure 1b). Metabolomic changes between weaning and 11.5 months of age are likely to be linked to growth and development, as the heifers’ diet and environment remained unchanged. Taken together, these data indicate distinct phases in bovine serum metabolome within the first 12 months of life. The sources of variation were initially targeted based on VIP scores. These indicated that up to 3 months the major changes were increases in 3-phenylpropionate and acetic acid (Figure 1c). Subsequently, up to 19 months of age, important changes appeared to be broadly linked to increases in some eicosanoids, with elevated levels of alpha-linoleic acid and eicosapentaenoic acid being particularly prominent (Figure 1d). Interestingly, acetic acid also accumulated during the early lactation negative energy balance (Figure S2).

**Figure 2:**
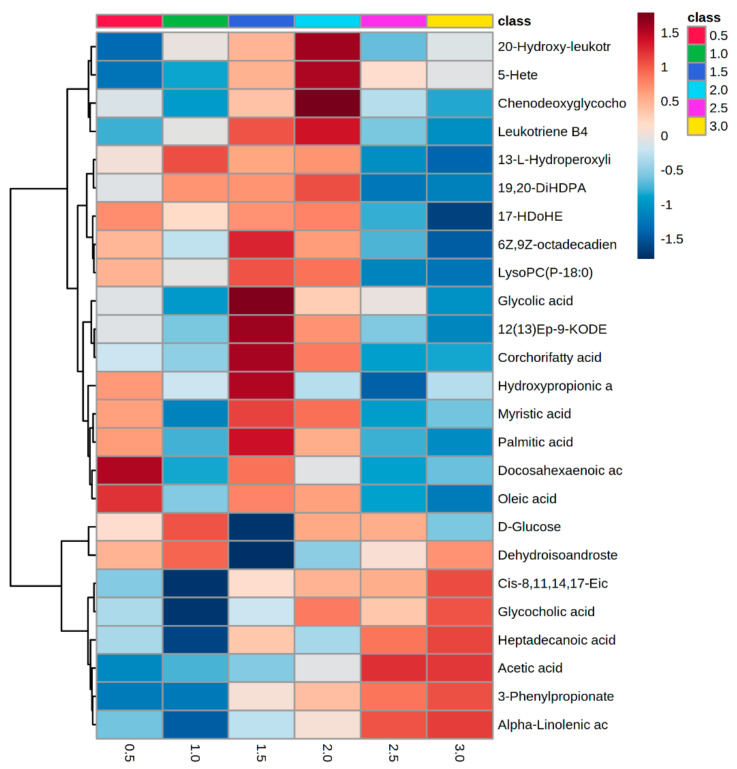
HCA of the major metabolite changes differentiating between HF heifers aged between 0.5 and 3.0 months of age in the combined ionization mode *m/z*.

**Figure 3:**
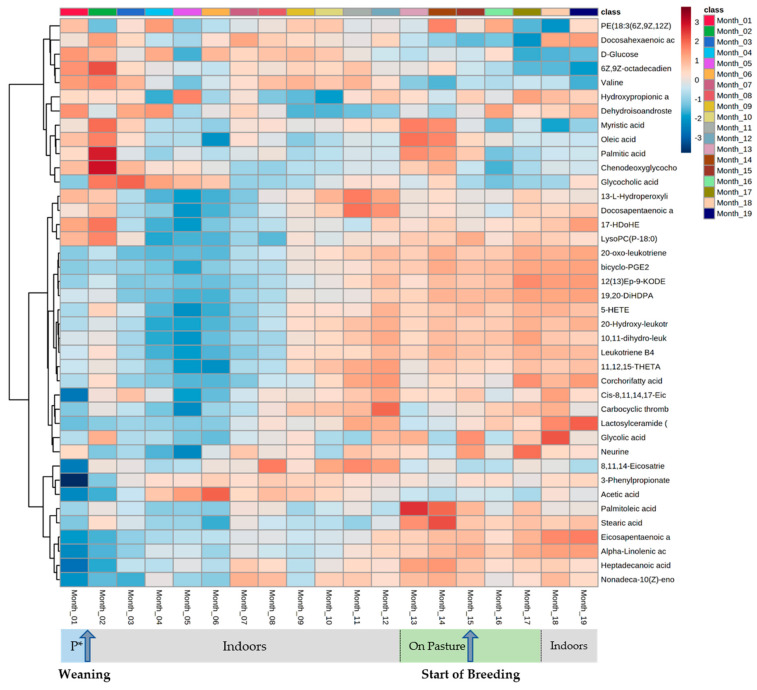
HCA of the major metabolite changes differentiating HF heifers aged between 0.5 and 3.0 months of age in the combined ionization mode *m/z*.

**Figure 4:**
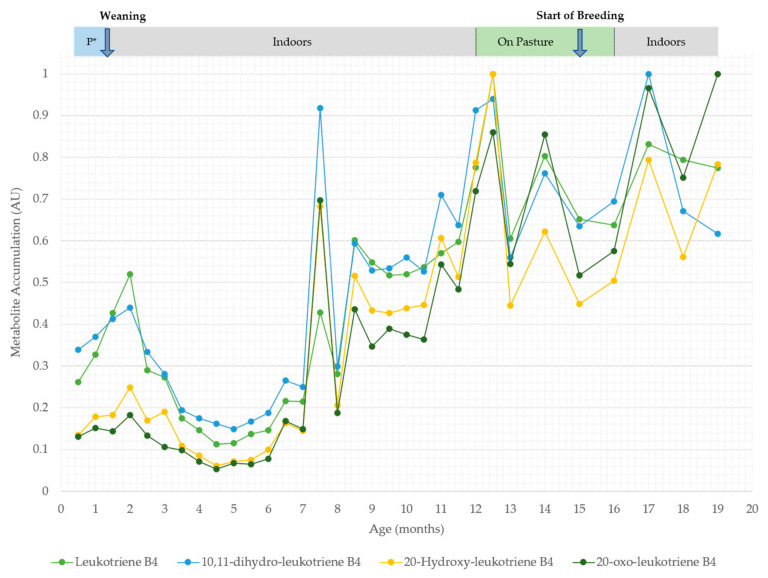
HCA of the major metabolite changes differentiating between HF heifers between 1 and 19 months of age in the combined ionization mode *m/z*. Changes in diet and/or environment are labelled accordingly, for example, P* = pre-weaning.

Then, *m/z* ratios, which differed significantly (*p*-values < 0.05, adjusted for FDR) between experimental classes, were targeted by time series analyses. The *m/z* ratios were identified and displayed using two heatmaps that displayed changes in the pre- and post-rumen development phases. Of the 30 metabolites identified between 2 weeks and 3 months of age, 20 metabolites displayed a peak in accumulation between 1.5 and 2.0 months of age (Figure 2) and changes in fatty acyl class metabolites were prominent (Table 1). Considering these in greater detail, the metabolites belonged to the following subclasses (number of metabolites): fatty acids and conjugates (11), linoleic acids and derivatives (4), eicosanoids (3) and fatty amides (1) (Table 1). Considering, metabolomic changes occurring post-rumen development, time series analyses targeted 40 metabolites (Figure 3). Of these, 22 metabolites increased, 13 decreased and 5 fluctuated with no clear pattern over time. Most of the metabolites were again fatty acyls (Figure 3), belonging to the following subclasses (number of metabolites): fatty acids and conjugates (15), eicosanoids (8) and linoleic acids and derivatives (4) (Table 1). Of the identified metabolites, 25 were also identified within 2 weeks to 3 months of age in the time series analysis (Table 1). Leukotriene-related metabolites (leukotriene B4, 10,11-dihydro-leukotriene B4, 20-hydroxy-leukotriene B4 and 20-oxo-leukotriene B4) generally increased over time but showed noticeable accumulation peaks at 2.0, 7.5, 12.5 and 17.0 months of age (Figure 4). The initial peak at 2.0 months of age broadly corresponds to weaning at approximately 6 weeks of age. Meanwhile, the peaks at 12.5 months and 17.0 months of age coincide with environmental and dietary changes. However, the underlying reason behind the isolated peak at 7.5 months remains unknown; it may be a technical anomaly, as it does not correlate with any known experimental variables. In line with this, pathway assessments based on MSEA using ORA indicated that only alpha-linolenic acid and linoleic acid metabolism significantly changed in pre- and post-weaning analyses (Figure S3).

**Figure 5:**
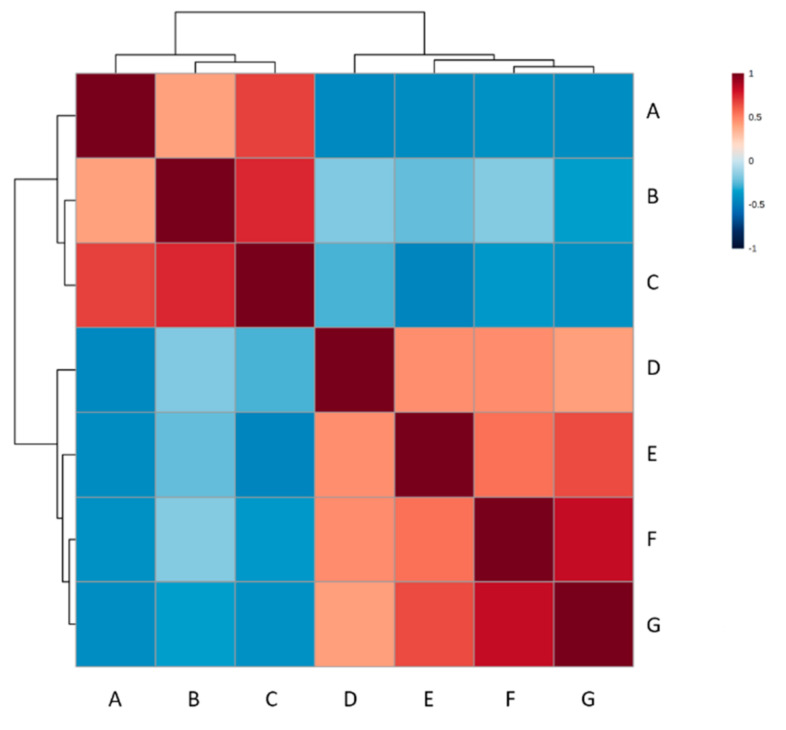
A heatmap of the Pearson’s correlation coefficients produced by comparing liveweight (kg) at 3.0 months of age and metabolites significantly affected by time between 2 weeks and 3 months of age, whereby −0.4 < correlation co-efficient > 0.4. Positive correlations are shown in red; negative correlations are shown in blue. A = liveweight (kg), B = 3-phenylpropionate, C = acetic acid, D = dehydroisoandrosterone 3-glucuronide, E = hydroxypropionic acid, F = 13-L−Hydroperoxylinoleic acid, G = docosapentaenoic acid.

Pearson’s correlation coefficient was used to assess whether the changes in any of the identified metabolites could be related to liveweight (kg) (Figures S4 and S5). Of 30 analyzed correlations between liveweight and metabolite profiles at 3 months of age, 2 were positively correlated (correlation coefficient > 0.4) and 4 were negatively correlated (correlation coefficient < −0.4). Acetic acid showed the strongest correlation, with a value of 0.679 (r2 = 0.46) (Figure 5 and Figure S4). This substantiated the importance of acetic acid in this first phase, as also indicated by its VIP score of 3.33 (Figure 1c). However, no metabolites showed a significant correlation (−0.4 < correlation coefficient > 0.4) with liveweight in the second phase (Figure S5).

## 5. Discussion

Rumen development, growth and sexual maturity are vital processes in bovine development and are crucial to the financial sustainability of dairy farms. Dietary factors, such as ration particle size and forage allowance, are known to influence rumen development [7] and ultimately affect liveweight. Nevertheless, balancing rearing costs, age at first calving and first-lactation milk production are vital for long-term profitability [18]. Advancements in the understanding of biochemical pathways active during the rearing phase which could be applied on-farm would benefit herd health and productivity, as well as improve financial outlooks for farmers. 

Previously, the utilization of omics approaches in dairy research has focused on lactating dairy cows. Metabolomic analysis has highlighted metabolites related to milk yield, milk composition and novel biomarkers for mastitis [9]. Additionally, multi-omics approaches have linked rumen microbial metabolites and specific microbial taxa [12,13]. However, few studies have applied metabolomic analysis to describe changes seen in vivo during heifer rearing. We used FIE-HRMS to demonstrate the importance of VFA in rumen development, followed by the role of fatty acyls, particularly eicosanoids, in weaning and growth. 

Although heifers were synchronized at 15 months of age, conception was staggered between approximately 15 and 17 months of age. Moreover, small and unequal small sample sizes resulted in the unfeasibility of time series analyses using two-way ANOVA aiming to identify pregnancy-related metabolites. Limited studies have examined how the bovine metabolome responds to pregnancy. Nevertheless, Phillips et al. [19] observed significant accumulations of amino acids (asparagine, lysine, glutamine, histidine, tryptophan, cysteine and ornithine) in fertile heifers. In contrast, Gomez et al. [20] observed lower levels of stearic acid and palmitic acid in successful pregnancies, including lower levels of valine between 40 and 62 days of gestation. Within our data, the accumulations of identified metabolites, including stearic acid, palmitic acid and valine, remained stable from 15 months of age (Figure 3), suggesting that synchronization and the subsequent gestation period had minimal impacts on the identified metabolites.

### 5.1. Serum Metabolomes Show the Influence of VFAs in Rumen Maturation

During rumen development, in response to increasing VFA concentrations, rumen pH decreases from approximately 6.0 to 5.0–5.5 [21], with possible effects on rumen epithelial development [7]. Indeed, with both pre-ruminant calves and mature cows, VFAs are released to decrease rumen pH and have a buffering effect [22]. Our metabolomic assessments clearly indicated the accumulation of the VFA acetic acid as the heifers were weaned and the rumen became functional (Figure 2). Interestingly, acetate increases were not associated with changes in other important VFAs—butyrate and propionate—in the sera. Instead, 3-phenylpropionate (3-PP) was shown to increase (Figure 2). 3-PP is a vital ruminal aromatic acid, accounting for 50.8% of total aromatic acids in the rumen fluid of forage-fed sheep [23]. Studies supplying varying levels of grain to lactating dairy cows reported positive correlations between acetate and 3-PP and rumen pH [22,24]. 

Interestingly, acetic acid and 3-PP were correlated (−0.4 > correlation co-efficient > 0.4) with liveweight (Figure 5). Previous studies have not directly compared VFA or ruminal aromatic acids with liveweight, but this association is implicit, as reticulorumen weight and liveweight exhibit a significant correlation [25]. As the rumen develops, the reticulorumen and abomasum occupy increasing and decreasing proportions of the forestomach (as a percentage of the total weight of the forestomach), respectively. The reticulorumen accounts for 38%, 61% and 67%, and the abomasum accounts for 49%, 25% and 15% of the forestomach in calves aged 0, 2 and 3–4 months of age, respectively [26]. Further, nutritional assessments have often investigated VFA levels alongside the rumen or liveweight. For example, Lin et al. [27] examined the effects of adding hay to the diets of pre-weaned calves at varying ages. Acetate and liveweight displayed similar patterns, but only liveweight was significantly affected by the addition of hay to the diet. Likewise, calves fed milk via the step-down method displayed increased rumen pH, acetate, propionate and liveweight when compared to conventional feeding methods [28]. Taking all of these factors together, the levels of serum acetic acid and 3-PP may indicate rumen maturation, reticulorumen weight and liveweight increases. 

### 5.2. Serum Oxylipids Indicate Inflammatory and Innate Immune Responses

Beyond VFAs, most of the changes in the serum metabolomes were associated with longer chain fatty acids. Similar changes are seen with weaning in a monogastric. A colonic metabolomic analysis of piglets pre- and post-weaning using gas chromatography tandem time-of-flight mass spectrometry (GC–TOF-MS) indicated changes in numerous fatty acids, including myristic acid, stearic acid and arachidic acid, and demonstrated a VIP > 1 [29]. Likewise, a comparison of hepatic metabolite profiles of weaned and un-weaned piglets using gas chromatography–mass spectrometry (GC–MS) showed elevated fatty acid metabolism, with phosphoethanolamine, ethanolamine and glycerophosphoglycerol being prominent [30]. Our more detailed longitudinal assessment indicated that many serum metabolites showed an increase between 1.5 and 2.0 months of age (Figure 2), which also coincided with the calf weaning period [31].

Weaning frequently induces an innate immune response, elevating proinflammatory cytokines [32], tumour necrosis factor-α (TNF-α) and cortisol levels but decreasing interferon-γ (IFN-γ) levels [33]. Crucially, 15 of our metabolites were fatty acyls (Table 1), often products of alpha linolenic acid and linoleic acid metabolism (Supplementary Figure S3), which have established roles in the regulation of inflammatory pathways [34]. These included omega-6 (n-6) and omega-3 (n-3) polyunsaturated fatty acids (PUFAs) that can promote or reduce inflammation. When required, PUFAs are released from membrane phospholipids and hydrolyzed by phospholipase enzymes to act as substrates for specific oxylipid synthesis [34]. Generally, inflammation is reduced by n-3 PUFAs (such as eicosapentaenoic acid and docosahexaenoic acid; Figure 3) and promoted by n-6 PUFAs (such as 5-HETE and leukotriene B4; Figure 3) [35]. The observation that both pro- and anti-inflammatory PUFAs were observed in the sera metabolomes was indicative of a marked modulation of inflammatory events as heifers were weaned and the rumen became functional. The increases in leukotriene B4 metabolites (LTB4, 10,11-dihydro-leukotriene B4, 20-hydroxy-leukotriene B4 and 20-oxo-leukotriene B4) could be indicative of wider immunological changes. Leukotriene B4 is synthesized following the release of arachidonic acid from membrane phospholipids [36] and interacts with various immune cells, including the promotion of IL-6 [37], IL-1 and TNF production by macrophages [38]. These proinflammatory cytokines have been shown to be elevated post-weaning [32]. Furthermore, nutritional and environmental changes are known to induce stress in lactating dairy cattle [39]. There is an absence of studies exploring the omics effects of indoor housing vs. pasture or grass silage vs. grass. Nevertheless, genes associated with four intermediate enzymes of leukotriene synthesis (ALOX5, ALOX5AP, LTA4H and LTC4S) have been shown to increase in abundance following a short-term subacute ruminal acidosis (SARA) challenge [40]. Along with our metabolomic data, this indicates the activation of the innate immune system response. 

If weaning and growth, alongside environmental and dietary changes, are associated with activation of the immune responses, a key question is how these could be subsequently suppressed. n-3 PUFAs simultaneously increase mitochondrial biogenesis, cell survival and growth but decrease inflammation by inhibiting eicosanoid production, subsequently boosting cell differentiation and aiding muscle repair [41]. This could be the role of the numerous n-3 PUFAs, for example, alpha linolenic acid and eicosapentaenomic acid, which are seen to increase from approximately 8 months of age (Figure 3). Such modulation of the immune responses may directly or indirectly be linked to n-3 PUFA growth-promoting properties. In support of this, HF calves receiving 40 g n-3 PUFAs per day for 61 days, from 13.7 ± 2.5 days of age, were reported to have significantly increased turnout weights and end weights [42]. Likewise, pre-weaned HF calves receiving 3–5 g/day of linoleic acid and 0.3–0.6 g/day of α-linoleic acid demonstrated improved growth [43]. Examining our data, we did not show a significant correlation between serum n-3 PUFAs and liveweights following weaning, although a significant positive correlation was observed between docosapentaenoic acid and liveweights at the pre-weaning stages (Figure 5).

## 6. Conclusions

Metabolomic analysis of HF heifers aged between 2 weeks and 19 months of age demonstrated the ability of untargeted FIE-MS to identify rumen development, weaning and growth-related changes. Time series analysis of calves between 2 weeks and 3 months of age, followed by correlation analysis between identified metabolites and liveweight, highlighted how acetic acid and 3-PP support rumen development and growth. Targeted metabolites suggest that weaning induces elevated levels of fatty acyls that reflect a post-weaning stress-induced innate immune response. Further, n-3 PUFAs could play a role in modulating immunity and promoting growth. Crucially, our work identified serum metabolites indicative of the calf maturation process that could be monitored to improve nutrition, health and growth. Future work could include extending our longitudinal sampling to include first calving, so metabolites associated with the transition period could be determined.

## Figures and Tables

**Table 1:** Metabolites which change over time in HF heifers aged 0.5 to 3 months and 0.5 to 19 months of age.

Class	Subclass	Metabolites	Mode	Age (Months)			
				0.5–3.0		0.5–19.0	
				F-Value	*p*-Value	F-Value	*p*-Value
Carboxylic acids and derivatives	Amino acids, peptides and analogues	Valine	Pos	-	-	1.31 × 10^1^	2.06 × 10^−48^
	Carboxylic acids	Acetic acid	Neg	1.69 × 10^1^	2.77 × 10^−11^	6.48 × 10^1^	1.72 × 10^−165^
Fatty acyls	Eicosanoids	20-Hydroxy-leukotriene B4	Neg	2.79 × 10^0^	2.55 × 10^−2^	4.21 × 10^1^	9.68 × 10^−127^
		5-Hete	Neg	1.05 × 10^1^	1.23 × 10^−7^	3.19 × 10^1^	2.97 × 10^−104^
		Leukotriene B4	Neg	5.24 × 10^0^	3.95 × 10^−4^	2.91 × 10^1^	2.02 × 10^−97^
		10,11-dihydro-leukotriene B4	Neg	-	-	2.00 × 10^1^	5.99 × 10^−72^
		11,12,15-THETA	Neg	-	-	1.68 × 10^1^	1.74 × 10^−61^
		20-oxo-leukotriene B4	Neg	-	-	4.21 × 10^1^	8.13 × 10^−127^
		bicyclo-PGE2	Neg	-	-	4.09 × 10^1^	1.93 × 10^−124^
		Carbocyclic thromboxane A2	Neg	-	-	7.86 × 10^0^	5.11 × 10^−28^
	Fatty acids and conjugates	11,14,17-Eicosatrienoic acid	Neg	-	-	2.47 × 10^0^	2.65 × 10^−5^
		12(13)Ep-9-KODE	Neg	1.11 × 10^1^	5.15 × 10^−8^	4.67 × 10^1^	1.19 × 10^−135^
		17-HDoHE	Neg	8.09 × 10^0^	4.06 × 10^−6^	1.27 × 10^1^	4.62 × 10^−47^
		19,20-DiHDPA	Neg	5.15 × 10^0^	4.54 × 10^−4^	2.73 × 10^1^	1.01 × 10^−92^
		9,10,13-TriHOME	Neg	4.45 × 10^0^	1.49 × 10^−3^	-	-
		Cis-8,11,14,17-Eicosatetraenoic acid	Neg	5.01 × 10^0^	5.77 × 10^−4^	3.89 × 10^0^	4.15 × 10^−11^
		Docosahexaenoic acid	Neg	8.02 × 10^0^	4.49 × 10^−6^	3.79 × 10^0^	1.12 × 10^−10^
		Docosapentaenoic acid	Neg	-	-	1.30 × 10^1^	3.97 × 10^−48^
		Eicosapentaenoic acid	Neg	-	-	2.32 × 10^1^	2.78 × 10^−81^
		Eicosenoic acid	Neg	7.42 × 10^0^	1.13 × 10^−5^	-	-
		Heptadecanoic acid	Neg	4.17 × 10^0^	2.41 × 10^−3^	9.65 × 10^0^	2.68 × 10^−35^
		Myristic acid	Neg	6.94 × 10^0^	2.41 × 10^−5^	9.50 × 10^0^	1.01 × 10^−34^
		Nonadeca-10(Z)-enoic acid	Neg	-	-	2.06 × 10^0^	7.96 × 10^−4^
		Oleic acid	Neg	6.16 × 10^0^	8.58 × 10^−5^	6.22 × 10^0^	4.23 × 10^−21^
		Palmitic acid	Neg	2.38 × 10^1^	1.49 × 10^−14^	1.25 × 10^1^	2.06 × 10^−46^
		Palmitoleic acid	Neg	-	-	3.74 × 10^0^	1.86 × 10^−10^
		Stearic acid	Neg	-	-	1.72 × 10^0^	9.85 × 10^−3^
	Fatty amides	Oleamide	Neg	7.38 × 10^0^	1.20 × 10^−5^	-	-
	Lineolic acids and derivatives	13-L-Hydroperoxylinoleic acid	Neg	7.75 × 10^0^	6.83 × 10^−6^	2.33 × 10^1^	1.05 × 10^−81^
		6Z,9Z-octadecadienoic acid	Neg	5.23 × 10^0^	4.00 × 10^−4^	5.61 × 10^0^	1.81 × 10^−18^
		Alpha-linolenic acid	Neg	3.60 × 10^0^	6.36 × 10^−3^	4.11 × 10^1^	9.48 × 10^−125^
		Corchorifatty acid F	Neg	9.28 × 10^0^	6.84 × 10^−7^	2.40 × 10^1^	1.22 × 10^−83^
Glycerophospholipids	Glycerophosphocholines	LysoPC(P-18:0)	Neg	1.11 × 10^1^	4.83 × 10^−8^	2.96 × 10^1^	9.10 × 10^−99^
	Glycerophosphoethanolamines	PE(22:6) ^1^	Neg	1.31 × 10^1^	3.14 × 10^−9^	-	-
	Glycerophosphoethanolamines	PE(18:3) ^2^	Neg	-	-	9.65 × 10^0^	2.53 × 10^−35^
Hydroxy acids and derivatives	Alpha hydroxy acids and derivatives	Glycolic acid	Neg	5.96 × 10^0^	1.18 × 10^−4^	4.84 × 10^0^	3.64 × 10^−15^
	Beta hydroxy acids and derivatives	Hydroxypropionic acid	Neg	5.64 × 10^1^	1.37 × 10^−24^	6.42 × 10^1^	1.52 × 10^−164^
Organonitrogen compounds	Quaternary ammonium salts	Neurine	Pos	-	-	5.80 × 10^0^	3.70 × 10^−19^
Organooxygen compounds	Carbohydrates and carbohydrate conjugates	D-Glucose	Neg	1.18 × 10^1^	1.96 × 10^−8^	2.11 × 10^1^	4.75 × 10^−75^
Phenylpropanoic acids	-	3-Phenylpropionate	Neg	2.94 × 10^1^	7.81 × 10^−17^	4.69 × 10^1^	3.73 × 10^−136^
Sphingolipids	Glycosphingolipids	Lactosylceramide^3^	Pos	-	-	2.17 × 10^1^	1.22 × 10^−76^
Steroids and steroid derivatives	Bile acids, alcohols and derivatives	Chenodeoxyglycocholic acid	Neg	8.62 × 10^0^	1.80 × 10^−6^	1.30 × 10^1^	4.11 × 10^−48^
		Glycocholic acid	Neg	7.27 × 10^0^	1.43 × 10^−5^	6.23 × 10^0^	3.85 × 10^−21^
	Steroid esters	CE(22:6) ^4^	Pos	3.20 × 10^0^	1.55 × 10^−2^	-	-
	Steroidal glycosides	DHEA 3-glucuronide	Pos	2.85 × 10^0^	2.74 × 10^−2^	2.33 × 10^0^	9.65 × 10^−5^

^1^ PE(22:6) = PE(22:6(4Z,7Z,10Z,13Z,16Z,19Z)/14:1(9Z)). ^2^ PE(18:3) = PE(18:3(6Z,9Z,12Z)/18:4(6Z,9Z,12Z,15Z)). ^3^ Lactosylceramide = Lactosylceramide (d18:1/16:0). ^4^ CE(22:6) = CE(22:6(4Z,7Z,10Z,13Z,16Z,19Z)).

## Data Availability

The metabolomics and metadata reported in this paper are available in the Supplementary Materials.

## Supplementary Materials

The following are available online at https://zenodo.org/record/6346831, Figure S1: PLS-DA of HF heifers between 0.5 and 19.0 months of age, in the combined ionization mode *m/z*, Figure S2: (**a**) PLS-DA, (**b**) VIP score plots and (**c**) HCA cluster analysis of two HF cattle serum sampled 1 month and 4 months post-partum during their first and second lactations. Abbreviations: 3-Hydroxybutyric a = 3-hydroxybutyric acid, salicylic acid bet = salicylic acid beta-D-glucoside, 1-(4Z,7Z,10Z,13Z,1 = 1-(4Z,7Z,10Z,13Z,16Z,19Z-docosahexaenoyl)-glycero-3-phosphate, LysoPE(0:0/22:4(7Z = LysoPE(0:0/22:4(7Z,10Z,13Z,16Z)), trimethylamine N = trimethylamine N-oxide, 7-Acetoxy-6-hydrox = 7-Acetoxy-6-hydroxylimonin and 8,11,14-Eicosatrie = 8,11,14-Eicosatrienoic acid, Figure S3: Significantly enriched pathways in HF heifers (a) between 2 weeks and 3 months of age and (b) between 2 weeks and 19 months of age. Both in the combined ionization mode *m/z*, Figure S4: A heatmap of the Pearson’s correlation coefficients produced by comparing metabolites significantly affected by time between 2 weeks and 3 months of age and liveweight (kg). Positive correlations are shown in red; negative correlations are shown in blue, Figure S5: A heatmap of the Pearson’s correlation coefficients produced by comparing metabolites significantly affected by time between 2 weeks and 19 months of age and liveweight (kg). Positive correlations are shown in red, negative correlations are shown in blue, Table S1: Raw data, negative ionization mode, Table S2: Raw data, positive ionization mode.
